# An integrated framework for examining groundwater vulnerability in the Mekong River Delta region

**DOI:** 10.1371/journal.pone.0292991

**Published:** 2023-10-20

**Authors:** Kathryn A. Powlen, Saira Haider, Kyle W. Davis, Nina Burkardt, Sachin Shah, Stephanie S. Romañach, Matthew E. Andersen

**Affiliations:** 1 U.S. Geological Survey, Oklahoma-Texas Water Science Center, Austin, TX, United States of America; 2 U.S. Geological Survey, Wetland and Aquatic Research Center, Davie, FL, United States of America; 3 U.S. Geological Survey, Nevada Water Science Center, Carson City, NV, United States of America; 4 U.S. Geological Survey, Office of International Programs, Reston, VA, United States of America; University of Parma: Universita degli Studi di Parma, ITALY

## Abstract

The Mekong River provides water, food security, and many other valuable benefits to the more than 60 million Southeast Asian residents living within its basin. However, the Mekong River Basin is increasingly stressed by changes in climate, land cover, and infrastructure. These changes can affect water quantity and quality and exacerbate related hazards such as land subsidence and saltwater intrusion, resulting in multiple compounding risks for neighboring communities. In this study, we demonstrate the connection between climate change, groundwater availability, and social vulnerability by linking the results of a numerical groundwater model to land cover and socioeconomic data at the Cambodia-Vietnam border in the Mekong River Delta region. We simulated changes in groundwater availability across 20 years and identified areas of potential water stress based on domestic and agriculture-related freshwater demands. We then assessed adaptive capacity to understand how communities may be able to respond to this stress to better understand the growing risk of groundwater scarcity driven by climate change and overextraction. This study offers a novel approach for assessing risk of groundwater scarcity by linking the effects of climate change to the socioeconomic context in which they occur. Increasing our understanding of how changes in groundwater availability may affect local populations can help water managers better plan for the future, leading to more resilient communities.

## Introduction

River deltas in many parts of the world are increasingly stressed from growing water demands, infrastructure development, and land cover change, as well as increases in the severity and frequency of extreme weather events linked to climate change [1, 2]. These events can affect water quantity and quality and drive additional changes such as land subsidence and saltwater intrusion, posing compounding risks to an estimated 600 million people who live in or near river deltas worldwide [3, 4]. To increase preparedness and the ability to respond, it is critical that we advance our understanding of the risks that these changes in water quantity and quality pose to local communities and their livelihoods. In this study, we demonstrate the effect of climate change on groundwater availability and social vulnerability in the Mekong River Delta region, a region undergoing rapid change and expected to become more vulnerable in the future [5–7].

Vulnerability assessments can be used to understand potential effects of climate change, land cover change, and infrastructure development on communities by examining *exposure*, *sensitivity*, and *adaptive capacity* [8–10]. Exposure refers to the degree to which a system or population will experience change and sensitivity refers to the degree to which it will be affected by that change [9]. Adaptive capacity is the ability of a population to absorb or respond to the change [9, 11]. A growing body of literature examines social vulnerability to climate change in the Mekong Region [12, 13] as well as vulnerability to specific shocks, such as floods [14–16]. However, slow-onset risks such as groundwater scarcity, driven by climate change and overextraction, have been examined less frequently. Given the critical role that groundwater plays in food security and meeting domestic water demands, in addition to its role in mediating other risks such as drought, land subsidence, and saltwater intrusion, a greater examination of groundwater variability and potential sensitivity is needed.

In recent years, it has become increasingly common for Mekong River Delta region residents to use groundwater for domestic, agricultural, and industrial purposes, especially throughout the dry season [17–19]. A 2015 study reported that there were more than 1 million groundwater wells in the delta and that the majority were private shallow-tube wells installed for household consumption [20]. More than one half of the households in Cambodia rely on groundwater as a primary source of drinking water, with an estimated 270,000 hand-pumped drinking water wells across the country [21]. In Vietnam, groundwater extraction has been estimated at approximately 1,924,000 m^3^/day with about 42% for domestic use, 40% for agricultural purposes, and 18% for industry [21]. However, groundwater is predicted to become less available in the region, as is true on a global scale, threatening food security and community resilience [3, 22].

In this study, we develop an integrated vulnerability framework to assess the future risk posed by groundwater scarcity on communities in the Mekong River Delta region along the Cambodian and Vietnamese border in 2040. We use a numerical groundwater model to estimate exposure to groundwater stress, or the potential decline in groundwater availability during 2020–40. We estimate sensitivity to that stress in relation to agriculture-related livelihoods by calculating agriculture, aquaculture, livestock, and domestic water demands. An existing adaptive capacity framework was used to understand how local populations will be able to respond to groundwater scarcity. In doing so, this study contributes to existing climate-risk research by presenting an approach that can be used to advance our understanding of the risk that groundwater scarcity poses on communities and livelihoods. The design and results of this study can be used to inform water managers and assist with future water-use planning.

### Theoretical framework and existing literature

**Fig 1:**
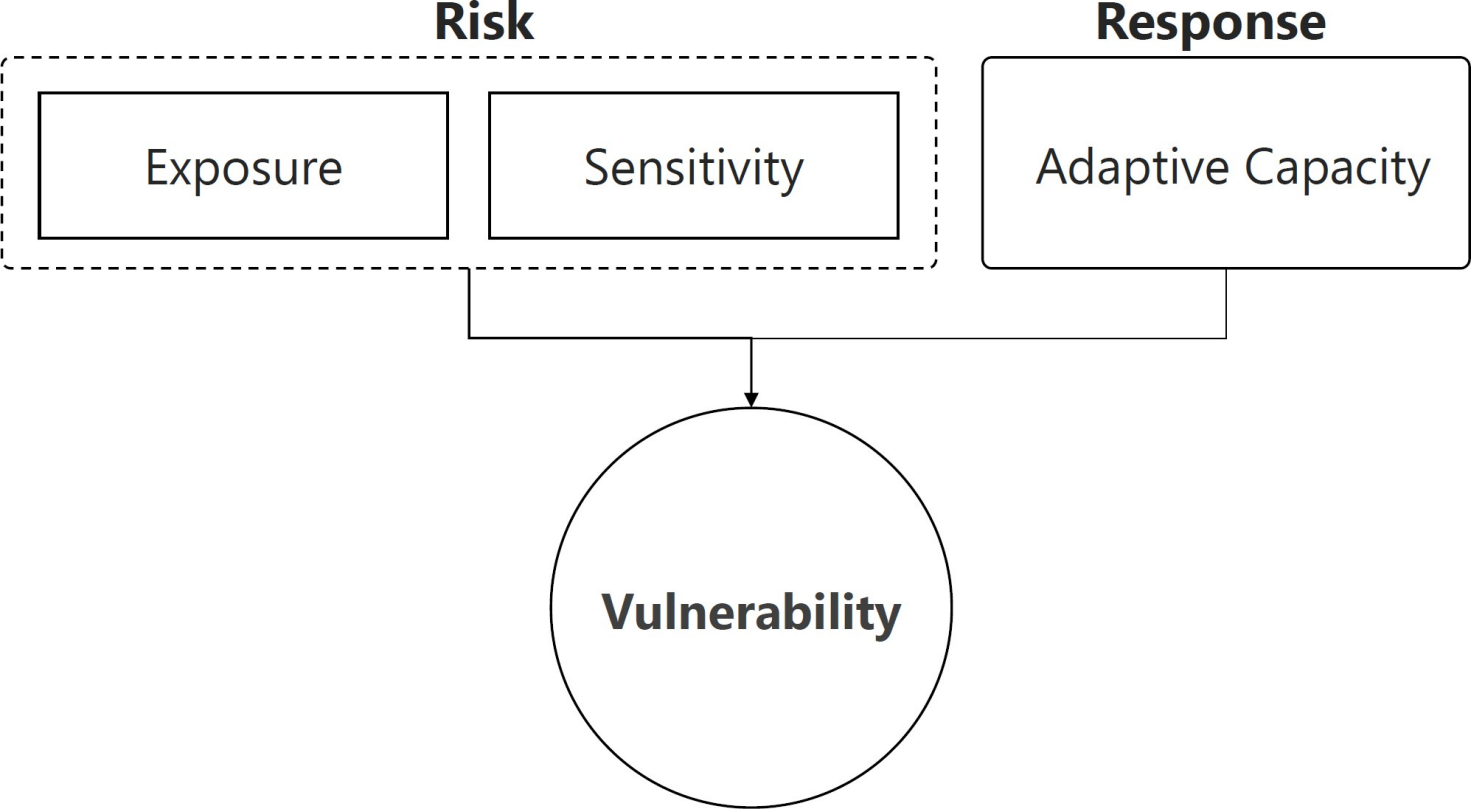
Vulnerability framework: Figure displaying key components of the vulnerability framework used in this study (adapted from [24]).

The concept of *vulnerability* has been adopted by various disciplines, leading to uncertainty in the origins of vulnerability research [8]. This has led to diverse applications of the concept and approaches used in vulnerability assessments [9]. For example, vulnerability has been examined as an outcome as well as an underlying context [23] and can be driven by a lack of resources or by exposure to natural hazards [8]. We include three main components of vulnerability in our assessment—exposure, sensitivity, and adaptive capacity—following the Intergovernmental Panel on Climate Change (IPCC; 2007) definition of climate change vulnerability, which states that vulnerability is “…the degree to which a system is susceptible to, or unable to cope with, adverse effects of climate change, including climate variability and extremes. Vulnerability is a function of the character, magnitude, and rate of climate variation to which a system is exposed, its sensitivity, and its adaptive capacity.” [24]. In doing so, we discuss exposure and sensitivity as the *risk* caused by groundwater scarcity and adaptive capacity as the *response* capabilities of communities (Fig 1). Greater adaptive capacity ultimately reduces vulnerability, leading to more resilient communities.

Existing vulnerability research in the Mekong River Delta region has focused primarily on floods [14–16, 25], with a few studies on drought [7, 26, 27] and climate change impacts more broadly [e.g., 12, 13]. These studies frequently compile multiple indicators identified as determinants of vulnerability into a single index, such as a household vulnerability index [13] or a livelihood vulnerability index [16, 26], using equal or weighted averages. Weighted averages can strengthen indexes by more accurately reflecting the level of importance of each indicator for adaptation but require additional data collection to accurately reflect these relationships [13, 14, 28]. Existing indices have examined dimensions such as socio-demographic characteristics, family structure, health, livelihood strategies, knowledge and skills, tenure status, social networks, housing means, and financial assets or credit.

In other vulnerability focused research in the Mekong region, qualitative methods have been used to identify factors that help communities adapt or cope with climate change risks [29–31]. For example, in three Vietnam provinces in the Mekong River Delta region, Le et al. (2014) found that adaptive capacity to climate change effects is determined by both resource constraints, such as tenure insecurity, lack of access to credit, and a lack of information on adaptation strategies, as well as psychological factors such as the perceived importance of adaptation [30]. Additionally, a 2018 study conducted within a 15 km corridor on either side of the Mekong River found that the most prevalent short-term strategies used to cope with effects of floods and drought were reliance on government assistance or employment-driven migration [31]. The authors also found shifting agricultural practices, such as using climate-resilient crop varieties and shifting planting calendars, were also important long-term strategies [31].

Literature examining vulnerability to groundwater stress has been much more limited, with only a few studies examining this link in Bolivia [32], Mexico [33], the United States [34], and at a global scale [22]. These studies often use socioeconomic data and the results of groundwater models to demonstrate the spatial variability of vulnerability. For example, Ojeda Olivares et al. (2020) combined population growth and marginalization with a groundwater model to identify populations vulnerable to groundwater stress in Oaxaca, Mexico. The authors found more than 50% of the study area was susceptible to water stress and identified groundwater extraction, pollution, and reduced recharge as the main drivers of vulnerability. In a global analysis, Döll (2009) used results from WaterGAP and the Human Development Index to identify regions that were potentially vulnerable to groundwater scarcity by 2050 under four different climate scenarios. Results of this study found that almost one-fifth of the total global population may experience a decrease in groundwater availability by 2050. However, the authors also found that the areas with the highest sensitivity to water stress may not necessarily experience the greatest decrease in water availability [22].

We take a similar approach as these existing groundwater studies by combining socioeconomic data with the results of a groundwater model to identify areas vulnerable to groundwater stress. We use a climate scenario defined by the Mekong River Commission that depicts a drier and warmer climate (see [35]) to simulate potential changes in groundwater availability and represent exposure across the study area. We define sensitivity as a population’s dependency on freshwater for domestic needs and water-dependent livelihoods, such as agriculture and livestock raising. To examine adaptive capacity, we modify an existing framework from Cinner et al. (2018) and estimate capacity in five domains: asset base, information and learning, flexibility, institutions, and agency.

Indicators representing asset base in the adaptive capacity framework include both financial resources and access to services, such as health care and electricity. As groundwater levels decrease, increased financial resources will be required to access groundwater [36] or to purchase alternative water sources. Access to services, such as a central water provider, can increase the likelihood that alternative water sources are available. Access to services such as health care and electricity increases the capacity to respond to health risks such as water contamination. Information and learning indicators reflect access to education and knowledge exchange opportunities which can increase adaptive capacity through more accurate risk perceptions [30] and by introducing more diverse livelihood opportunities [29]. Flexibility indicators reflect the choices and opportunities available for individuals to adapt in response to a shock or stress [11]. For example, occupation driven migration can reflect a lack of alternative livelihood opportunities, and ultimately, low adaptive capacity. Institution indicators reflect access to organizations and institutions that provide supporting services or resources, and agency reflects the ability to leverage those resources. Memberships in formal or informal organizations and access to technical experts can provide opportunities for social learning which can increase awareness of risk and increase innovation, ultimately leading to higher adaptive capacity [37]. Finally, we focus on gender-based agency, given that previous research has found women are often more vulnerable than men to climate change effects in the Mekong River Basin [31]. To examine agency, we use an existing gender equality index [38], which is compiled from multiple indicators such as male-to-female ratios in education, labor force, and political positions. The five adaptive capacity domains are not mutually exclusive, and a change in one domain can often influence another [11].

## Methods

### Study area

**Fig 2:**
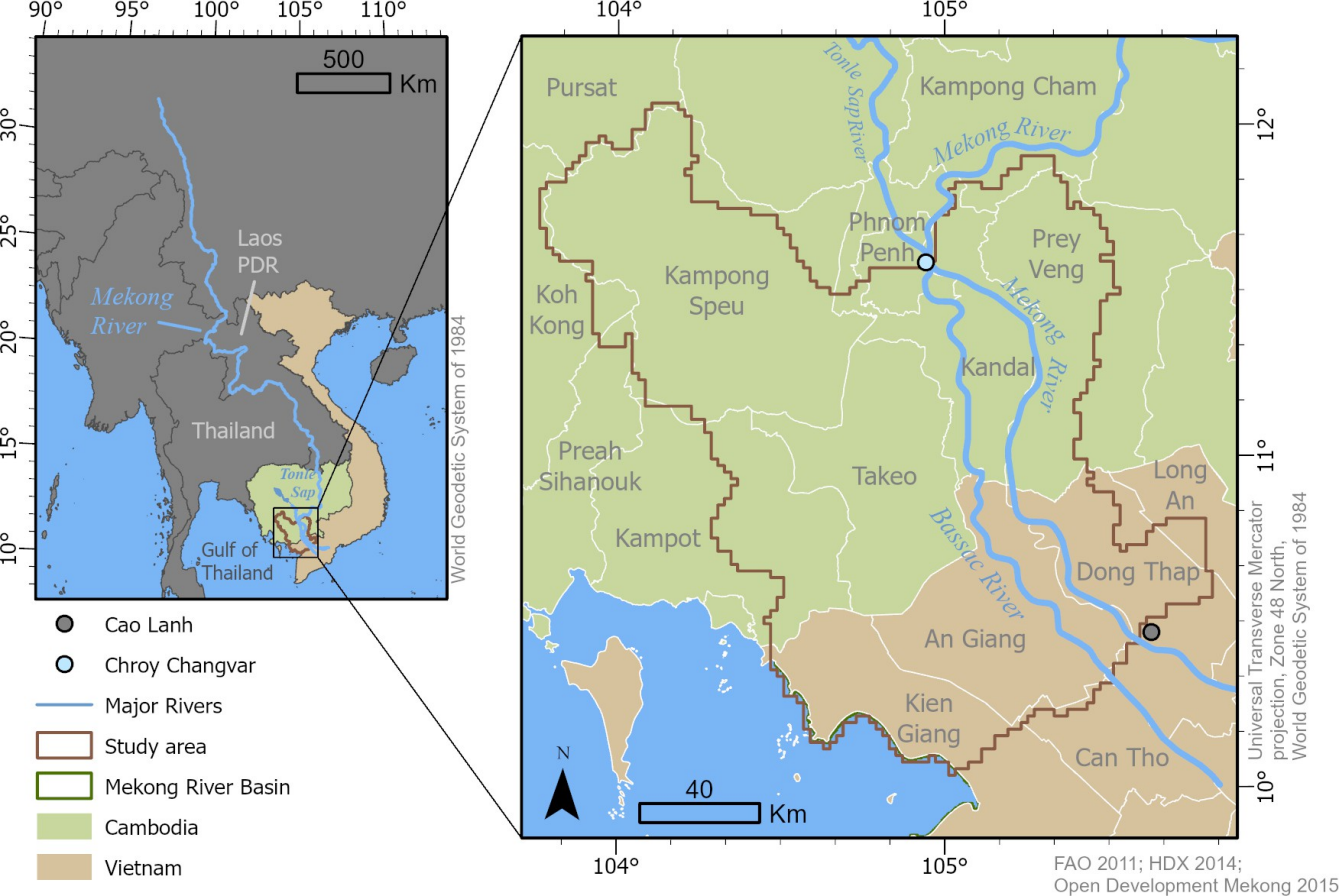
Map of the study area.

The Mekong River stretches across six countries, starting in the Tibetan Plateau (where the river is known as the Lancang River) and flowing through China, Myanmar, Laos, Thailand, Cambodia, and Vietnam, where it empties into the sea (Fig 2). Although the river is critically important for biodiversity and the communities that rely on it, it is becoming increasingly altered by hydropower infrastructure, expanded agriculture, increased navigation, sand mining flood protection, increased domestic and industrial water use, and climate change [35]. Together, these forces are projected to change hydrologic flow patterns and water availability [39, 40] and reduce sediment deposition and fishery productivity [35]. Recent climate change projections predict an increase in temperature [41] and in hydrologic cycle extremes, including increases in streamflow during high-flow events [39], potentially leading to increased flood risk. Other studies have reported an increase in drought occurrence in the region, with drought frequency, especially flash droughts, expected to increase in the future [7, 42]. Hydropower developments are also changing historic hydrologic flow patterns, lowering rainy season streamflow and increasing dry season base flows [40, 43]. Potential effects in the Mekong River Delta region of Vietnam are among the most extreme, as this part of the Mekong Basin is projected to undergo additional risks such as sea level rise, salinity intrusion, and land subsidence [6, 40, 44].

In addition to driving changes in the natural functioning of the river, development and climate change will affect employment opportunities and food security for local populations. A large majority of the people living in the Mekong River Delta region engage in primary sector livelihoods, including agriculture, fisheries, and forestry [45]. The Mekong River Delta region has also undergone rapid growth in aquaculture driven by increased salinity and market forces [45]. However, recent (2021) trends in employment in the primary sector diverge between Cambodia and Vietnam. For example, primary sector employment in Cambodia is estimated at 66% of the total population, compared to Vietnam, which is estimated at 41% of total population [45].

A report from 2021 suggests that at the national level both countries produce sufficient food to meet domestic dietary requirements [45] and Vietnam is currently one of the world’s top rice-exporting countries [46]. However, the future of food security is threatened by changes to the Mekong River [7, 45, 46]. Both rice production and fish catch are predicted to decrease with ongoing dam development and climate change unless adaptation measures are implemented [43, 46–48]. Throughout the Mekong River Delta region, it remains to be seen how climate change and changes in land cover and infrastructure development will interact, either to counteract or amplify impacts, resulting in high uncertainty for future conditions.

For this study, we selected a transboundary area that coincides with the 6-digit hydrologic unit code watershed boundary of the lower part of the Mekong River Basin approximately between Phnom Penh, Cambodia and Cao Lanh, Vietnam (Fig 2). The study area is centered over the Cambodian and Vietnamese border and covers 25,575 km^2^. The study area overlaps with 10 provinces in Cambodia and 5 in Vietnam but does not contain the full area of each province because the study area follows watershed, not political boundaries to meet groundwater modeling requirements. Including provinces from two neighboring countries provides an opportunity to investigate whether international neighbors experience different levels of groundwater stress and sensitivity to that stress. To our knowledge, this is the first study examining social vulnerability to groundwater stress in the Mekong River Delta region.

### Data and analysis

#### Exposure: MODFLOW model

We defined exposure as the potential stress that an aquifer may undergo as a result of a changing climate. We estimated exposure using simulated water-level differences from a proof-of-concept MODFLOW 6 groundwater model [49]. In our proof-of-concept model, the Mekong Delta aquifer system and underlying bedrock in the study area were represented using a simplified hydrogeologic framework, consisting of six layers each with unique thicknesses and hydrologic properties, and with simplified representations of hydrologic processes based on hydrologic data for a transient, 20-year period [35, 40, 50–52]. The grid resolution was selected to be approximately equivalent to 0.1 degree in the World Geodetic System 1984 (WGS84) coordinate system resulting in 2,200 by 2,200 m grid cells. This simplified model can be used to demonstrate how a system may differentially respond to variations in climate conditions based on biophysical characteristics. However, the hydrogeology and other model inputs can be refined in the future to create an independently robust tool that water managers can use to assess groundwater availability in the study area.

The proof-of-concept model simulated groundwater flow for parts of the Mekong Delta aquifer system and underlying bedrock aquifers and used a routed streamflow process to simulate streamflow and stage for segments of the Mekong and Bassac Rivers in the study area for combined steady-state and transient periods (Fig 2). First, baseline conditions and groundwater levels were simulated for a 20-year period between November 1990 and October 2010. The 20-year baseline period was selected primarily based on the availability of stream stage records for the Mekong River near Phnom Penh at the Chroy Changvar stream gage in Cambodia [53]. The end of the 20-year period was assumed to approximately represent recent (2020) groundwater conditions in the study area, and the end of this period provided baseline transient groundwater levels to calculate exposure (i.e., change in groundwater levels). The model was then modified to represent a drier climate, similar to one of the Mekong River Commission’s (MRC’s) climate scenarios (C3) [35], for another 20-year period representing potential groundwater conditions for the period of 2020 to 2040. The transient outputs from the end of this scenario simulation were compared to the baseline simulation to calculate potential groundwater stress.

Streamflow and recharge were specified for a steady-state period (pre-November 1990) and for transient periods that were three months long between November 1990 and October 2010, representative of baseline conditions. The steady-state period at the start of the simulation was used to provide initial conditions for the 20-year transient simulation period. Both the dry and the wet seasons were represented by two transient stress periods per year to approximately differentiate between flood and drought within a 12-month period: (1) dry season *A* (Nov. 1–Jan. 31), (2) dry season *B* (Feb. 1–Apr. 30), (3) wet season *A* (May 1–Jul. 31), and (4) wet season *B* (Aug. 1–Oct. 31) [54]. A maximum potential evapotranspiration rate of 1.65 meters per year and an extinction depth of 2.5 meters [50] were assigned uniformly to the entire simulation period. Streamflow input for the baseline simulation was based on streamflow calculated by using a rating curve for the Chroy Changvar stream gage in Phnom Penh and was the mean streamflow during each stress period [53]. Recharge was applied by using spatially uniform rates equivalent to an assumed 16% of precipitation in Cambodia for each stress period [51] and was approximately consistent with recharge used in a groundwater model for Kampong Speu province, Cambodia [50].

Recharge and streamflow inputs for the scenario model were determined based on estimates of monthly precipitation for 2040 from the MRC’s C3 scenario and were averaged to be representative of the three-month long stress periods. Recharge was lower in both the dry and wet seasons compared to baseline conditions in the scenario model [40]. Streamflow for the C3 dry scenario was lower in the wet season and typically higher in the dry season compared to the baseline scenario [52], likely as a result of upstream hydropower development. The percent change from baseline conditions for recharge and streamflow was linearly interpolated from the start of the simulation period to the end of the simulation period and was applied to each model stress period. Additionally, 0.21 meters of sea-level rise was linearly applied over the simulation period from 2020 to 2040 from 0 to 0.21 to the groundwater boundary representing the sea [35]. A complete description of the proof-of-concept groundwater model is available in the S1 File, and the model is available in an accompanying data release [53].

Exposure was calculated by subtracting transient groundwater levels in the uppermost majority non-dry model layer for each stress period in the C3 scenario model from the equivalent layer and stress periods in the baseline model. However, some cells in the model did contain dry baseline estimates, which resulted in a small number of cells with missing data in the exposure and vulnerability calculation. All simulated water level changes reflect those driven only by climate and other biophysical factors, such as landscape or aquifer characteristics, given that groundwater withdrawal data were not available and therefore, not included in the model. The groundwater level differences were reported for the end of a transient stress period for the model layer from which the baseline water levels were obtained as a percentage of the baseline layer saturated thickness (in meters) multiplied by the layer hydraulic conductivity (in meters per day [m/d]) divided by 50 m/d, the highest value of hydraulic conductivity assigned to a layer in the model. Dividing by the highest hydraulic conductivity value highlights groundwater level changes in areas where groundwater is more likely to be used as a water source and where most wells exist in the study area and de-emphasizes groundwater level changes in areas of lower hydraulic conductivity, where groundwater is less likely to be used. This approach effectively highlights the changes in transmissivity of the uppermost aquifer in the model area under drier climatic conditions. Normalized transient groundwater level changes were calculated for the end of the last wet and dry seasons of the C3 climate scenario; however, only the results of the dry season are presented in this manuscript. Results for the wet season are included in the supplementary material.

#### Sensitivity

We defined sensitivity as the reliance on freshwater for domestic and agriculture-related livelihoods because a majority of the population in the Mekong River Delta region engages in primary sector livelihoods. Our sensitivity calculation assumed a “business-as-usual” scenario, holding water demands for domestic and agriculture-related livelihoods consistent across the 20 years. We used secondary data sources to estimate current water use because data on water extraction were not available. More specifically, we used census data, various global and regional geospatial data, and water consumption rates to estimate domestic, agriculture, aquaculture, and livestock water demands (see [55] for more information on methods and data sources).

*Domestic water needs*. Water requirements for human populations were estimated using census data and a human population dataset from WorldPop.org (see [56]). Province-level rural and urban human population counts were gathered from the Cambodian General Population census [57] and the Vietnamese Population and Housing census [58]. Annual urban and rural water consumption per capita estimates by country were gathered from existing literature for the most recent date available (see Table 1). Consumption rates for urban and rural regions were multiplied by their respective population counts for each province to calculate a weighted province-level domestic water use estimate. The 2019 unconstrained population count datasets from WorldPop.org, created in partnership with the Geo-referenced Infrastructure and Demographic Data for Development (GRID3) using methods outlined in [56], were used to distribute the weighted province-level water demands at a 100 m resolution for both Cambodia and Vietnam.

**Table 1:** Water consumption rates. Annual rates of water requirements for domestic, livestock, agriculture, and aquaculture.

*Domestic*	*Water Consumption (m*^*3*^*/yr*.*)*	*Source *
Individual in urban Vietnam	47.5	[59]
Individual in urban Cambodia	36.5	[60]
Individual in rural Vietnam	29.2	[61]
Individual in rural Cambodia	25.5	[62]
*Livestock*	*Water Consumption (m*^*3*^*/yr*.*)*	*Source*
Buffalo	18.25	[63]
Cattle	16.43	[63]
Chicken and duck	0.11	[64]
Pig	5.50	[63]
*Agriculture & Aquaculture*	*Water Consumption (m* ^*3*^ */ha)*	*Source *
Rice - wet season/dry season	7,215/6,122	[65]
Maize	6,120	[65]
Sugar cane	14,623	[65]
Vegetables (used for cassava)	7,101	[65]
Fruit trees	18,592	[65]
Aquaculture	4,800	[63]

*Agricultural and aquacultural water needs*. Water requirements for agriculture and aquaculture were compiled by combining census and land cover data. We recorded the total planted hectares of the most common crops from the Cambodian [66] and Vietnamese censuses [67, 68], which included wet and dry season rice, maize, cassava, sugar cane, banana, mango, and coconut. We then used crop water consumption estimates (Table 1) to calculate the annual volume of water required to produce these crops by province. To distribute the agricultural water needs across each province, we used SERVIR-Mekong’s 2018 land cover maps generated from the Regional Land Cover Monitoring System (RLCMS) at 30-meter resolution [69]. We used four SERVIR-Mekong land cover categories—rice, cropland, planted forest/orchard, and aquaculture—to spatially distribute the water needs in each province. The final agricultural water needs estimate is equal to the sum of the water requirements for the planted area of the seven crops and aquaculture areas.

*Livestock water needs*. Livestock water requirements were estimated by using a combination of global livestock density data and recent census records. Cattle, duck, chicken, and pig density estimates were gathered from Livestock Geo-Wiki at a 1-kilometer resolution [70] and updated with more recent livestock numbers available from the 2019 Cambodia census [66] and 2019 Vietnam census [67]. The update was done by converting the Geo-Wiki livestock density estimates to the total numbers of livestock per province, per type of livestock, and multiplying by a ratio of the per province change in total livestock numbers to estimate 2019 total number of livestock per grid cell. The total number of buffalo were calculated by using census data exclusively because Livestock Geo-Wiki did not include buffalo densities. Annual volume of water required for each grid cell was calculated by multiplying the total number of each livestock by an estimate of water consumption per head (Table 1). The final livestock water needs estimate includes the sum of all buffalo, cattle, pig, chicken, and duck water requirements.

#### Adaptive capacity

To understand how communities are able to respond to groundwater stress, we compiled a list of adaptive capacity indicators and established predicted relationship based on a review of relevant literature. Indicators with a positive relationship are expected to increase adaptive capacity, ultimately reducing vulnerability, and those with a negative relationship are expected to decrease adaptive capacity, increasing vulnerability. For indicators to be included in this assessment, adaptive capacity indicators had to be available for both countries and have a consistent relationship to adaptive capacity across both countries. Variables such as poverty rate, access to internet, and tenure security were not included because they were not available for both countries and access to credit was removed because of variability in its relationship to adaptive capacity.

The final list consisted of 17 individual indicators organized into five domains of adaptive capacity, including asset base, information and learning, flexibility, institutions, and agency (Table 2). The gender inequality indicator categorized under agency, however, is itself an index gathered from the Gender Equality Monitoring SERVIR-Mekong. The index includes country-relevant indicators such as male-to-female ratios in education, labor force, political positions, and indicators on women’s health (see [38] for more details). The index includes seven indicators for Cambodia and nine for Vietnam.

**Table 2:** Adaptive capacity indicators. This is a list of indicators for adaptive capacity and their relationship with vulnerability organized by respective capacity domain.

*Adaptive Capacity Domain*	*Indicator*	*Relationship*
Asset Base	Labor force as % of population	(+)
	Employment rate	(+)
	Malnutrition under 5 years	(−)
	Birth life expectancy	(+)
	Electrification rate	(+)
	Access to sanitation services	(+)
	Access to central water provider	(+)
Information & Learning	Literacy rate	(+)
	Secondary education	(+)
	Trained worker rate	(+)
Flexibility	% income from agriculture	(−)
	Markets per capita	(+)
	Net migration	(+)
Institutions	% membership in cooperative, farmer assoc.	(+)
	Access to extension agents	(+)
	Access to bank	(+)
Agency	Gender inequality index	(−)

Data for each adaptive capacity indicator were collected at the province-level for both countries due to this being the finest scale available from the Cambodian census reports. The values were standardized by country to account for slight differences in census indicator measurement. We calculated a score for each of the domains individually (i.e., asset base, information and learning, flexibility, etc.) and a full adaptive capacity score for each province using the arithmetic mean [55]. All indicators were given equal weights within each domain in the compiled indices.

#### Vulnerability

We used estimates from the exposure, sensitivity, and adaptive capacity components above to calculate a vulnerability score. Each of the three components were reclassified on a scale of one to five to combine exposure, sensitivity and adaptive capacity into a single value. For exposure, the percent change in groundwater levels under the C3 climate scenario as compared to baseline conditions were grouped into five bins, representing +2.5% to 0%, -0.1% to -1.0%, -1.1% to -5.0%, -5.1% to -10.0%, and -10.1% to -100% and respectively reclassified to one through five, so that the higher value reflected higher exposure. For sensitivity, we also created five bins for the total annual water demands across the three uses (domestic, agriculture and livestock) which included 0 to 50,000m^3^, 50,001 to 100,000m^3^, 100,001 to 150,000m^3^, 150,001 to 200,000m^3^, and greater than 200,000m^3^. These bins were also reclassified to one through five where the higher value reflects higher sensitivity. For adaptive capacity, we used an equal interval to divide the index values into five classes, where higher values reflect higher adaptive capacity. The vulnerability score is calculated as exposure plus sensitivity minus adaptive capacity.

## Results

### Risk

**Fig 3:**
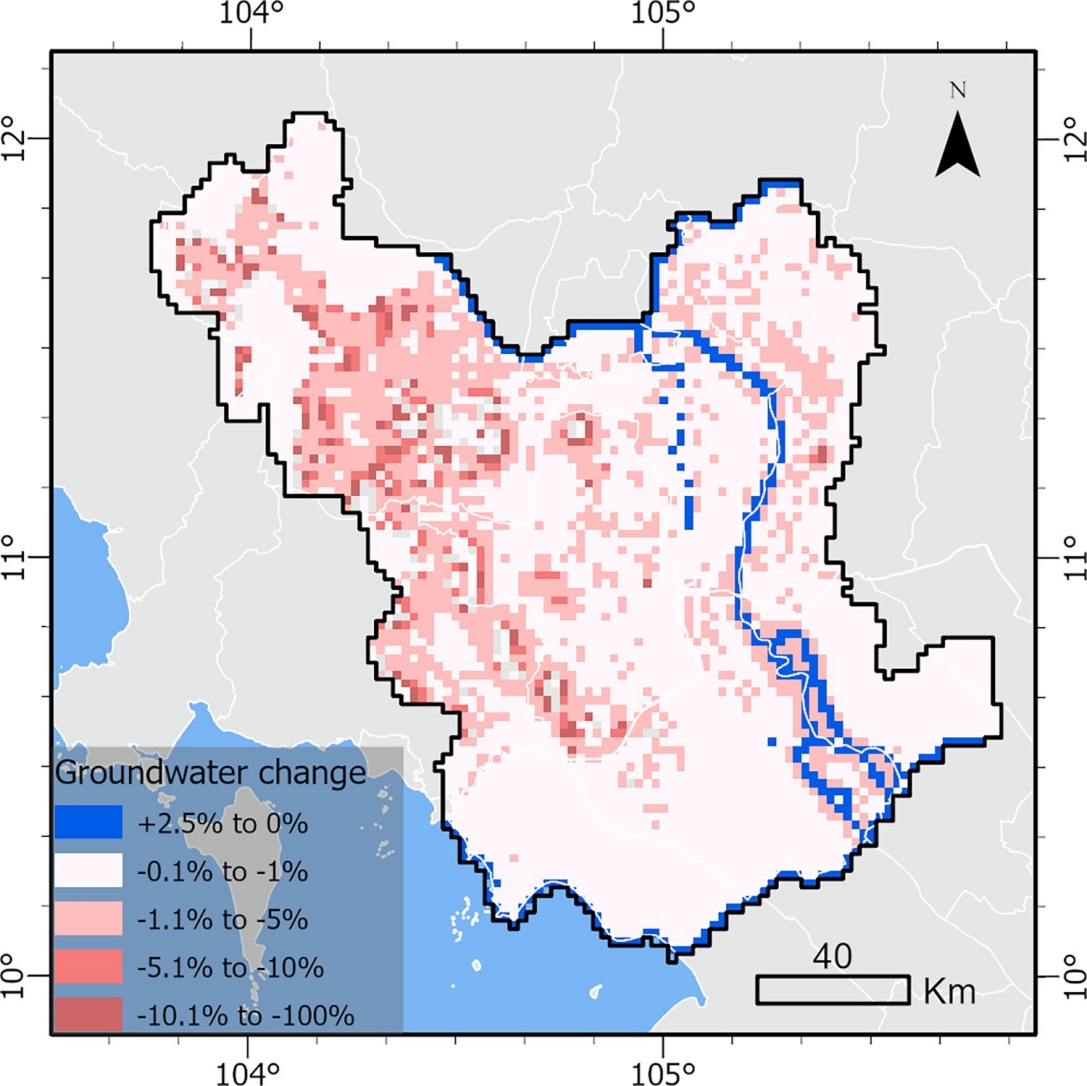
Exposure. Map of exposure to groundwater stress across the study area, calculated as percent change in groundwater levels under the simulated scenario compared to baseline conditions.

The proof-of-concept MODFLOW 6 model found varying degrees of exposure, or groundwater level changes, across the study area (Fig 3). The normalized groundwater level changes were grouped into five categories for interpretation purposes, including increases (+) between 0% to 2.5%, and decreases (−) between 0% and 1%, 1% and 5%, 5% and 10%, and greater than 10%. Groundwater level changes in the 0 to −1% and −1% to −5% categories represent areas where climate stress may minimally affect groundwater resources and where groundwater level changes are potentially recoverable from season to season or in wetter years. Groundwater level changes in the −5% to −10% and greater than −10% categories indicate areas where climate stress could substantially affect groundwater resources, to the point of them potentially becoming irrecoverable.

For most of the study area, simulated normalized groundwater level declines were between 0 and −5%. The greatest normalized groundwater level declines were in the northwestern part of the model area, specifically in the delta and alluvial aquifers in Kampong Speu, Takev, and Kampot provinces in Cambodia (Fig 3 and S1 Fig). These areas were often found to have declines greater than 1% of the initial saturated thickness compared to baseline conditions, potentially due to the altitude variation in these regions. Additionally, areas with groundwater level declines greater than −5% were only found in Cambodia.

In the southern part of the study area, groundwater level declines were generally between 0 and −1% of the baseline saturated thickness, with few areas experiencing −1 to −5%. The Mekong Delta region in Vietnam has much less land-surface relief and a much stronger hydraulic connection to the sea, Bassac River, and Mekong River compared to the northern part of the study area in Cambodia. This strong groundwater-surface water connection potentially led to smaller groundwater level declines in our model in this part of the study area. Areas found to have increased groundwater levels were directly influenced by sea level rise or the increased streamflow model input drawn from the MRC’s C3 scenario.

The predicted level of sensitivity, or degree of freshwater dependence, also varied across the study area. The greatest annual water demand for the combination of domestic, agricultural, and livestock categories was found in An Giang province in Vietnam, and the lowest demand was found in Koh Kong province of Cambodia. The predominant water use type varied across provinces and across countries. For example, more urbanized provinces such as Phnom Penh had higher water demands for domestic uses, whereas provinces such as An Giang, Dong Thap, or Can Tho had higher agricultural water demands. In general, we found higher agricultural water demands in the Vietnamese provinces of our study area and slightly higher livestock water demands in the Cambodian provinces. Overall, total water demands for the three categories were found to be much greater in Vietnam compared to Cambodia.

**Fig 4:**
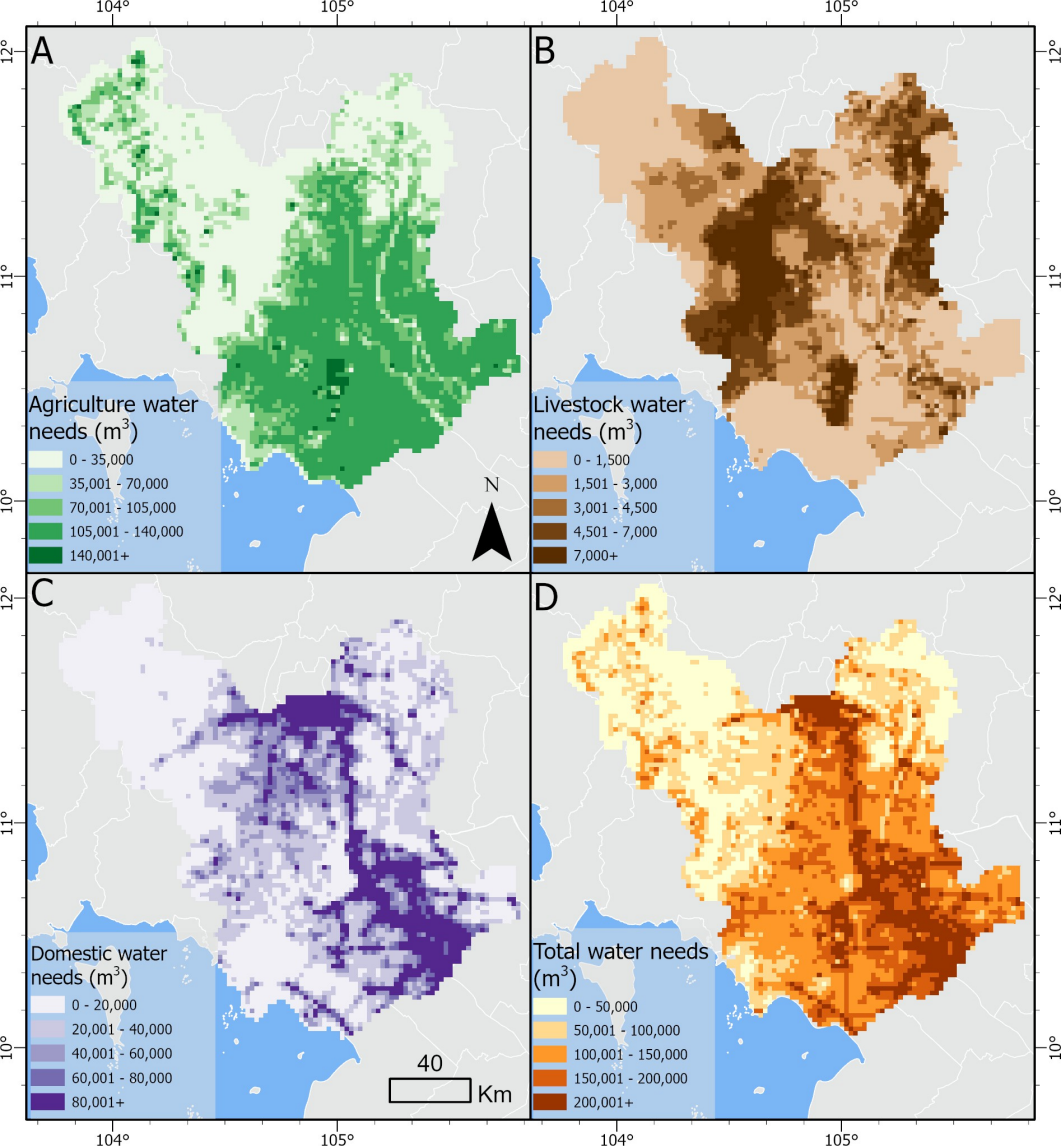
Water demand. Maps of water demands (m^3^/year) across the study area by use categories for A, agricultural water needs, representing water demands for rice, cropland, planted forest/orchards, and aquaculture; B, livestock water needs, representing water demands for buffalo, cows, pigs, ducks, and chickens; C, domestic water needs, representing water demands for urban and rural human populations; and D, total annual water needs, representing the sum of A, B, and C.

By spatially distributing the water demands using the land cover and human population geospatial datasets, we can identify patterns of water use across the study area (Fig 4A–4C). Agricultural water needs were largely concentrated in the southern region of the study area, where rice paddy fields are most common (Fig 4A). Additionally, discontinuous orchard patches were found in the northwestern region of the study area and in central An Giang province, increasing agricultural water demands in these areas. We find the areas with the greatest population density—thus the greatest domestic water demands—are located along the Mekong River (Fig 4C). This includes the north-central region of the study area, around Phnom Penh, and the southern region of the study area within the provinces of An Giang and Dong Thap. Agricultural water usage primarily drove the total water needs pattern (Fig 4D), followed by domestic water demands (Fig 4C). Livestock water needs per km^2^ were significantly lower than both (Fig 4B).

**Fig 5:**
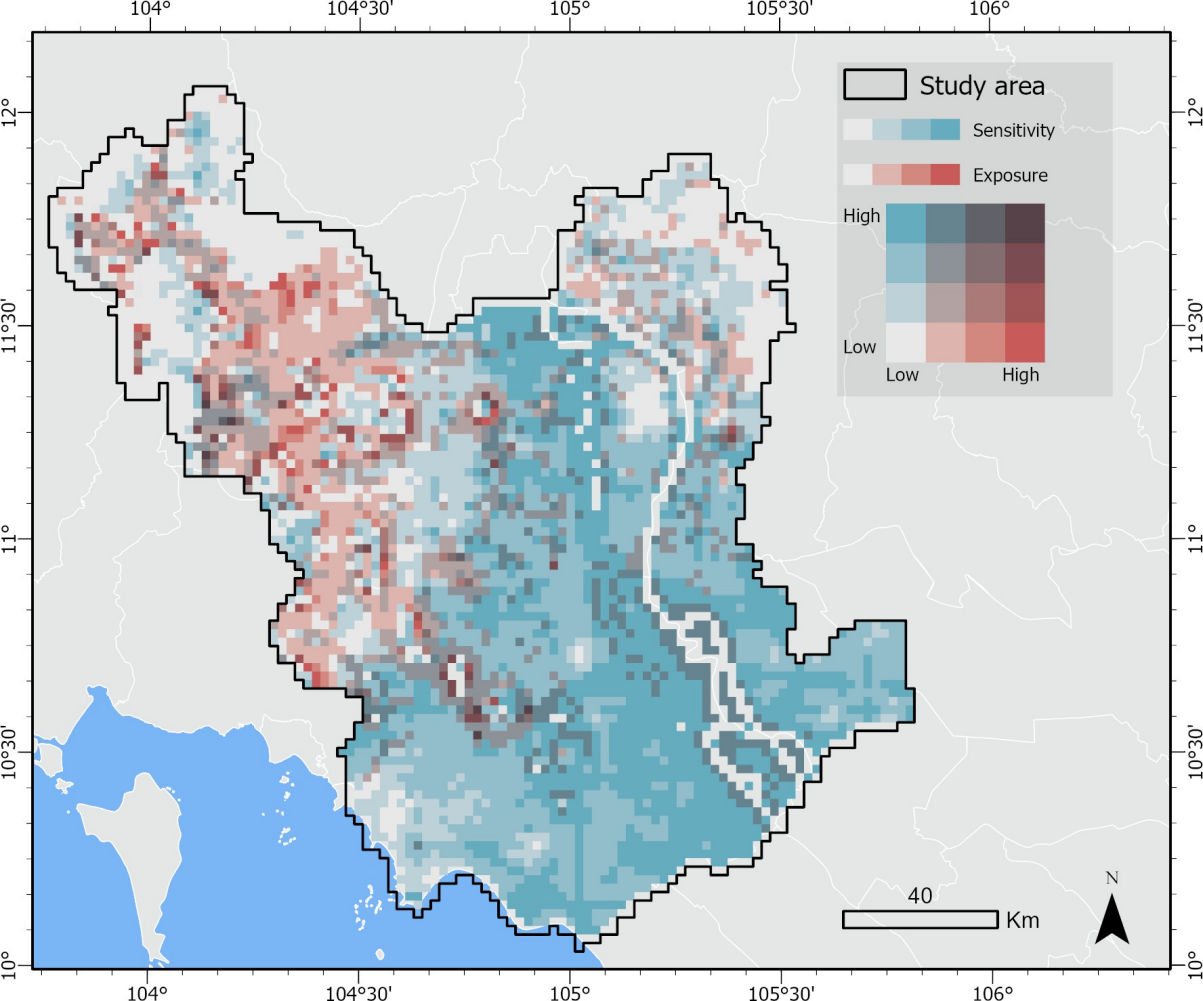
Risk map. Bivariate map showing risk of water stress based on exposure and sensitivity estimates. Exposure (i.e., MODFLOW 6 model outputs) is displayed in red and sensitivity (i.e., total water needs estimate; Fig 4D) is displayed in blue. Areas shown in purple have the greatest exposure and greatest sensitivity, and thus may experience the highest risk of water stress.

A bivariate map overlaying the exposure and sensitivity estimates identifies the areas with the greatest risk of water stress. Fig 5 displays exposure (i.e., MODFLOW 6 model outputs) in red and sensitivity (i.e., total water demand estimate) in blue. Areas shown in purple have the greatest exposure and greatest sensitivity, and thus may experience the highest risk of water stress. We find areas with the highest risk to be scattered in the west and northwest corner of study area, in the Takeo and Kampong Speu provinces, as well as along the river in the Vietnamese provinces of Dong Thap and An Giang, where we found high population density and rice farming (see Fig 2 for province boundaries).

### Response

**Fig 6:**
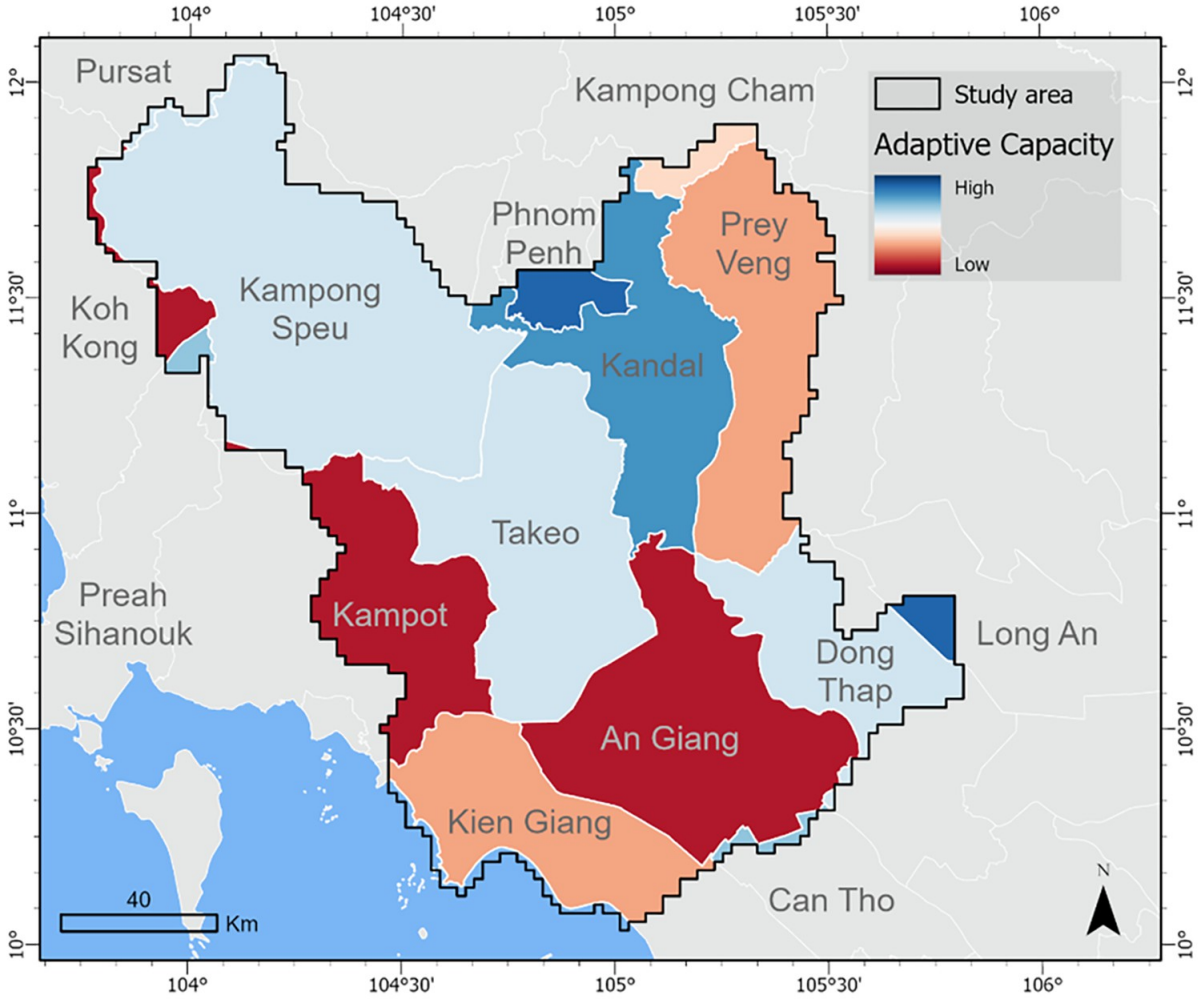
Adaptive capacity. Map of the adaptive capacity score calculated at the province level. The index is comprised of 17 indicators, which represent 5 capacity domains: asset base, information and learning, flexibility, institutions, and agency.

**Fig 7:**
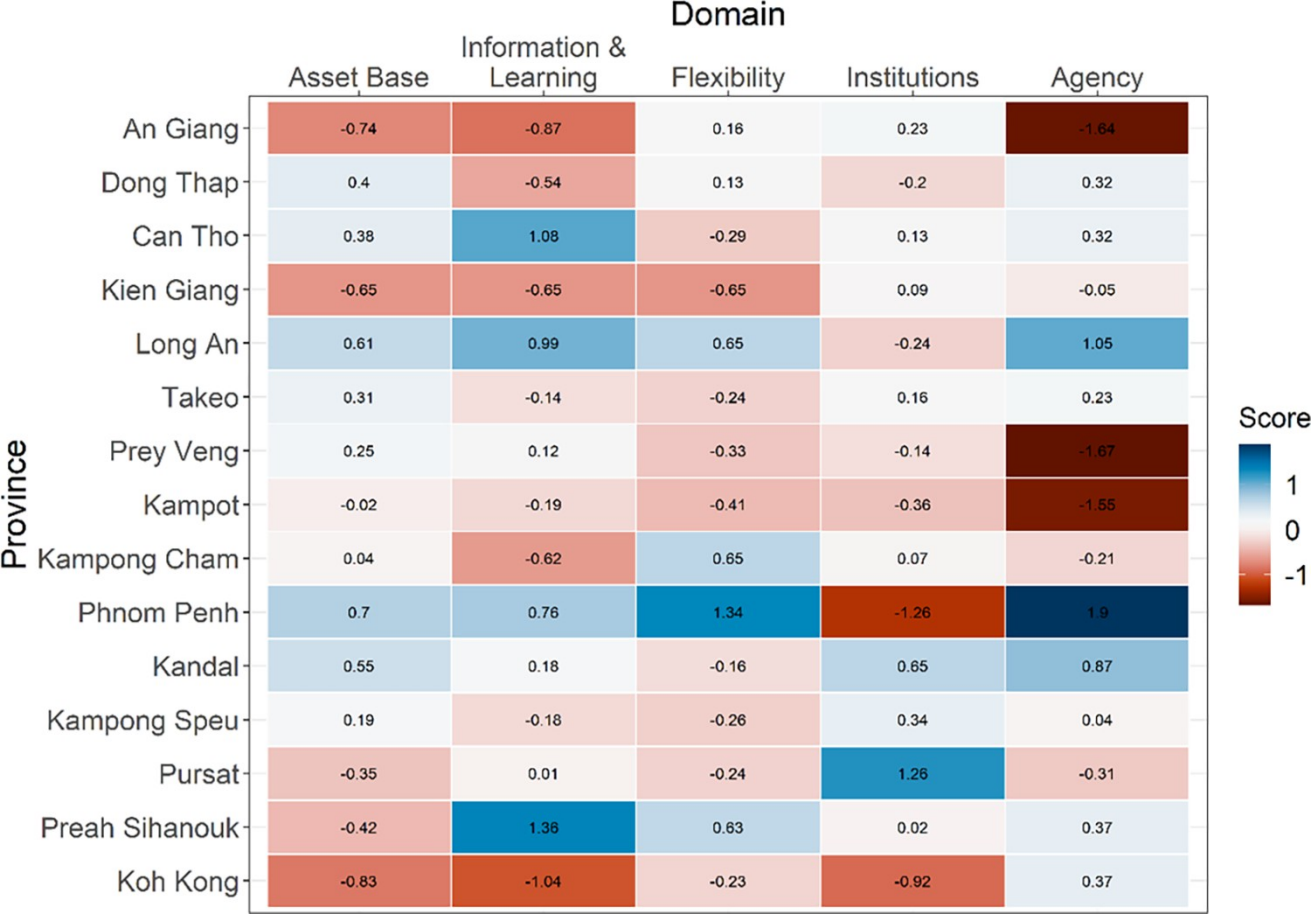
Components of adaptive capacity. Heat map of the adaptive capacity domain scores (range: -1.67 to 1.9) across all provinces.

Our adaptive capacity analysis found the highest response capabilities to groundwater stress in Phnom Penh and Long An, followed by Can Tho, Kandal, and Preah Sihanouk (Fig 6). Phnom Penh and Long An were found to have low institutional capacity because the indicators in this domain category reflect institutional support for agricultural activities (Fig 7). However, the high levels of capacity in the remaining four domains led to relatively high adaptive capacity scores for both provinces compared to the rest of the study area. Provinces with the lowest adaptive capacity estimates include An Giang, Koh Kong, and Kampot.

### Vulnerability

**Fig 8:**
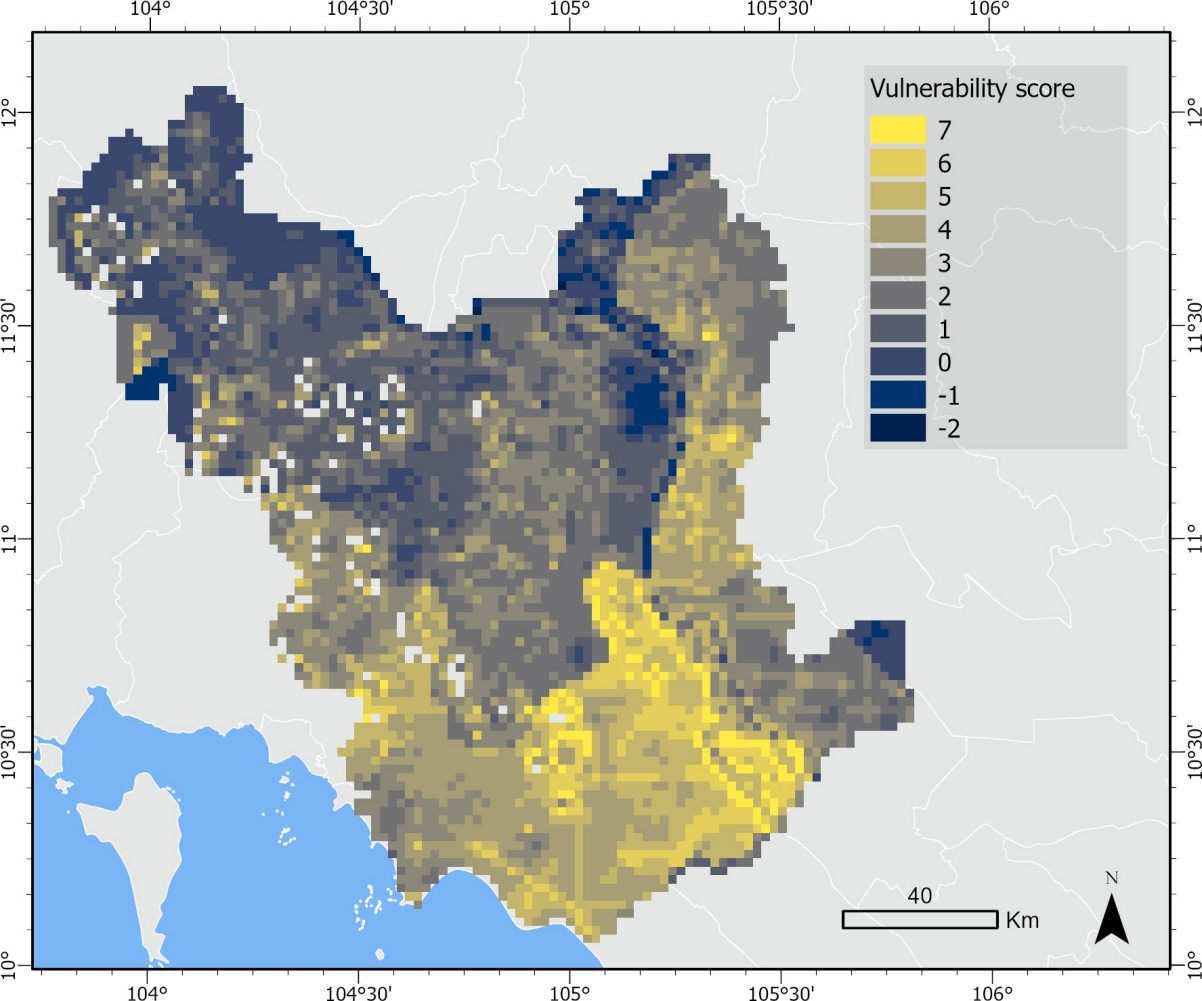
Vulnerability map. Vulnerability score calculated as exposure plus sensitivity minus adaptive capacity (range: -2-7). Higher vulnerability scores indicate greater vulnerability to groundwater stress.

Combining exposure (Fig 3), sensitivity (Fig 4) and adaptive capacity (Fig 6) provides a more holistic understanding of vulnerability to groundwater stress. Fig 8 shows the combined vulnerability score calculated as exposure plus sensitivity minus adaptive capacity. Areas with the highest risk of water stress and the lowest ability to respond are expected to be most vulnerable. For example, we find areas of high risk in the Vietnamese provinces of An Giang and Dong Thap; however, we find a greater concentration of vulnerability hotspots in An Giang compared to Dong Thap due to a lower adaptive capacity score in An Giang. Although An Giang had similar flexibility and better access to supporting institutions compared to Dong Thap, the province ranked lower in agency, asset base, and information and learning, indicating that An Giang may be more vulnerable to groundwater stress under drier climate conditions in the future. Additional vulnerability hotspots can be found in the Cambodian provinces of Koh Kong and Kampot along the western border of the study area and Prey Veng in the northeast.

## Discussion

Groundwater has become an increasingly important resource providing water for domestic and irrigation purposes in the Mekong Delta [17] and globally [22]. However, our understanding of the sustainability of groundwater as a primary water source is limited due to lack of data and information, as well as climate change uncertainty [71]. We help fill this knowledge gap by developing an integrated vulnerability framework aimed at gaining a holistic understanding of social vulnerability to groundwater stress, and we demonstrate its utility in the Mekong Delta. Our results show how the level of risk of water stress can vary within a transboundary watershed, as well as the ability of the local population to respond. Our results highlight that areas of high exposure (i.e., greater groundwater level declines) may not always overlap with areas that are most sensitive, which emphasizes the importance of examining exposure to natural hazard risks within the broader socioeconomic context in which they occur. This can help to better inform natural resource management and to more effectively develop interventions aimed at building resilience to climate change.

Our framework combines two common approaches for assessing vulnerability previously highlighted by Tran et al. (2017)—a modeling approach for assessing physical aspects of vulnerability and an indicator-based approach used to assess socioeconomic factors influencing vulnerability. By overlaying the results of our proof-of-concept groundwater model, which considers climatic and biophysical drivers of groundwater stress, with water demand estimates, which represent the socioeconomic drivers of sensitivity, we can better understand the extent of groundwater stress risk. In doing so, we were able to distinguish the areas that may experience high exposure and high sensitivity for prioritization. This is not to suggest that areas with alternative characteristics (e.g., high exposure and low sensitivity, low exposure and high sensitivity) should be ignored, but rather they may require comparatively less urgent attention for resource management interventions.

Indicator-based approaches to assessing vulnerability often condense multiple dimensions of vulnerability or capacities into a single index [9]. Although an indicator-based approach has proven to be a useful approach for gaining a broad understanding of vulnerability, its use masks important details that can help inform policy and decision-making [72]. Our approach to assessing adaptive capacity as multiple capacity domains overcomes this limitation and allows us to better understand what makes certain provinces more vulnerable than others. For example, the heat map in Fig 7 indicates that the most efficient approach to increasing resiliency in Koh Kong and Kampot might be through increasing access to supporting institutions, whereas An Giang may benefit more from increasing the technical capacity and knowledge transfer opportunities.

Patterns in our adaptive capacity assessment indicate that urbanized provinces may be more resilient to future water stress. This aligns with existing research in the Mekong region examining vulnerability to climate change more broadly [12]. Mai et al. (2016) links urban areas to higher resilience given their better access to services such as central water providers and health services, often higher financial asset bases, as well as increased diversity in livelihood opportunities. We also find the urban areas in our study area to be closer in proximity to the Mekong River, which could influence the accessibility of surface water or result in shallower, more accessible groundwater, increasing resilience. This study specifically examines vulnerability to changes in groundwater availability and does not consider issues related to water quality. Previous research has found groundwater in the Mekong Delta can often contain high levels of arsenic, iron, manganese, and fluorides, as well as high levels of salinity, making it unsuitable for domestic and irrigation purposes [21, 62]. Thus, an important future direction for this research would be to integrate data on groundwater quality to account for variability in groundwater suitability for the uses examined in this study.

Issues of scale can lead to tradeoffs in vulnerability and adaptive capacity research. Highlighting how drivers of household vulnerability and community vulnerability differ, Lam and Stringer (2018) argue household vulnerability is influenced by social assets, human assets, natural assets, financial assets, physical assets, and livelihood diversification, whereas governance and institutional capacity can influence community vulnerability [29]. We assess adaptive capacity at the province level to provide more detail and show greater variability across adaptive capacity indicators compared to a country-level analysis. In doing so, data that are only available at the country level, such as good governance indicators, were not included in the assessment despite potentially having an influence on adaptive capacity. Moreover, province-level statistics can still overlook spatial variation in factors such as access to services, employment opportunities, and education. To examine more accurately adaptive capacity and identify potential interventions to increase resilience, future research could downscale this part of the analysis to a finer resolution as additional data become available.

We overcome the lack of available data on groundwater extraction and water use by using secondary sources to estimate potential water demands for domestic, agriculture, and livestock purposes. We then use these estimates as proxies for the potential level of reliance on groundwater. We recognize that groundwater may not be used to fulfill all freshwater demands and that other water sources such as rainwater, surface water, or purchased water sources that are bottled outside the study area will contribute to the supply. Additionally, access to irrigation systems will vary across the study area. However, fieldwork limitations due to the COVID-19 pandemic and the lack of available data on groundwater extraction limited our ability to determine accurate ratios of water use by water source. Another potential improvement to the groundwater model could be made by incorporating calibrated hydraulic properties and enhanced aquifer geometry to measure the effects of sea level rise more accurately. This study demonstrates an approach to assess vulnerability in a data-scarce environment; however, future research could continue to refine the water use estimates and increase the amount of observed groundwater withdrawals used as data become available to better understand sensitivity, as well as gather relevant data to weight adaptive capacity indicators to better inform groundwater management decisions.

Finally, we focus on vulnerability relative to domestic, agriculture, and livestock-related livelihoods assuming stable water demands across each use sector. Given the dynamic nature of population change, land cover patterns, and global markets, it is likely that water demands will change during the next 40 years. Future research will be able to build upon the approach outlined in this paper by developing probable population and land use scenarios to understand vulnerability under varying conditions. Our framework could be adapted to assess the vulnerability of different industries within the same area or expanded to assess groundwater stress in other geographical regions. When applying this approach to new contexts, researchers could adjust the adaptive capacity indicators to the study area context, as well as “ground-truth” the predicted relationships through additional data collection with local populations [72], as the influence of indicators may vary regionally.

## Conclusion

As groundwater levels decline, groundwater will become more challenging and expensive to access in the Mekong Delta region, ultimately reducing its reliability as an alternate freshwater source. This will affect food security and domestic water use patterns, as well as drive additional challenges such as land subsidence and saltwater intrusion, all with high potential for negative consequences on the local populations. Here we present an integrated framework to increase our understanding of the influence of climate change on groundwater resources and how that will in turn affect local populations and water-dependent livelihoods. We demonstrate how risk to water stress varies based on the level of exposure and sensitivity to changes in freshwater availability. Finally, we use an adaptive capacity framework to assess how communities would be able to respond to increased water stress. Our approach provides a holistic assessment of vulnerability to groundwater stress and can be used to identify key strategies for building resilience to climate change impacts on water resources in the region.

## Supporting information

S1 File Details on numeric groundwater model. (DOCX)

S1 Fig Proof-of-concept groundwater model extent and grid cells linked to specific MODFLOW Packages. (DOCX)

S2 Fig Recharge applied to the baseline proof-of-concept groundwater model, calculated using 16% of precipitation for Cambodia. (DOCX)

S3 Fig Input streamflow at Phnom Penh, Cambodia for the baseline proof-of-concept groundwater model. (DOCX)

S4 Fig Relative groundwater level changes after 20 years of drier climate (C3) with sea-level rise for the A, wet season and B, dry season. Positive values indicate areas of groundwater level declines and negative values indicate increased groundwater levels.
(DOCX)

S1 Table Summary of hydrogeologic units in the groundwater model. (DOCX)

S2 Table Monthly percent change in recharge and streamflow applied to the end of the 20-year C3 climate scenario (Mekong River Commission, 2019), drier climate with sea level rise. Positive values indicate an increase from baseline.
(DOCX)
