# Supplementary material for: An integrated framework for examining groundwater vulnerability in the Mekong River Delta region
Source: PLoS One. 2023 Oct 20;18(10):e0292991. doi: 10.1371/journal.pone.0292991 (PMC10588840; doi:10.1371/journal.pone.0292991)
Supplement: S1 File — (DOCX) [file pone.0292991.s001.docx]

Supporting Information for

An integrated framework for examining groundwater vulnerability

in the Mekong River Delta region

Kathryn A. Powlen^1*^, Saira Haider^2^, Kyle W. Davis^3^, Nina Burkardt^4^, Sachin Shah^1^, Stephanie S. Romañach^2^, Matthew E. Andersen^4^

^1^U.S. Geological Survey, Oklahoma-Texas Water Science Center, Austin TX, United States of America

^2^U.S. Geological Survey, Wetland and Aquatic Research Center, Davie FL, United States of America

^3^U.S. Geological Survey, Nevada Water Science Center, Carson City NV, United States of America

^4^U.S. Geological Survey, Office of International Programs, Reston VA, United States of America

^*^Corresponding author

**Corresponding Author Information**

Address: U.S. Geological Survey, Oklahoma-Texas Water Science Center, 1505 Ferguson Ln, Austin, Texas 78754

Phone: 508-361-3455

Email: [kpowlen@usgs.gov](mailto:kpowlen@usgs.gov)

**S1 Text: Details on numeric groundwater model.**

A proof-of-concept MODFLOW 6 groundwater model [1] was constructed to provide a basis for linking a groundwater model and socioeconomic model in the study area, and it is not intended to be used to represent localized hydrologic processes or changes in hydrogeology. The proof-of-concept groundwater model is simplified in its representation of subsurface hydrogeology and the hydrologic processes that can influence groundwater levels, streamflow, or groundwater availability in the study area; however, it can be used to show how a system may differentially respond to variations in model inputs, primarily those that represent stresses from a changing climate. The hydrogeology and other model inputs can be refined in the future to create an independently robust tool that water managers can use to assess groundwater availability in the study area.

The proof-of-concept groundwater model was developed to represent the bedrock, delta, and alluvially deposited aquifers underlying the Mekong River Basin approximately between Phnom Penh, Cambodia and Cao Lanh, Vietnam (Fig S1). The model was constructed using square grid cells measuring 2,200 meters (m) on each side, which was selected to be approximately 0.1 degree in the World Geodetic System 1984 (WGS1984) coordinate system. The northwest corner of the model was located at 1,342,500 m North and 362,700 m East in the WGS84, Universal Transverse Mercator (UTM) zone 48 North geospatial projection.

The model was used to simulate baseline groundwater conditions for a steady-state period, representing conditions prior to November 1990, and transient conditions for November 1990 through October 2010 (water years 1991–2010). This period was selected based on available stream stage data for the Mekong River near Phnom Penh at the Chroy Changvar stream gage, Cambodia [2]. The end of the baseline simulation period (2010) was used to approximately represent current (2020) groundwater conditions. A second model (scenario simulation) was used to simulate potential groundwater conditions in the study area for a hypothetical climate scenario 20-years into the future (i.e., 2040). The hypothetical 20-year transient climate scenario uses characteristics from the Mekong River Commission’s (MRC) C3 climate scenario - drier climate with sea level rise - from the MRC Council Study Report [3]. The steady-state stress period was identical in the baseline and scenario simulations and was included to provide initial conditions for the transient stress periods of the models. Both models are available in an accompanying ScienceBase data release [2].

The Mekong River, its tributaries, and its distributaries generally overlay the alluvial and deltaic sediments of the Mekong River delta in the study area [4] and were assumed to be in hydraulic connection with the underlying aquifers. The intermittent sand, gravel, and silt deposits that make up the Mekong Delta serve as aquifers or aquitards depending on the local hydrogeology, and the bedrock units do not typically constitute aquifers in the study area. Multiple sequences of transmissive and non-transmissive materials likely exist in the Mekong Delta [4]; however, the hydrogeology in the study area was significantly simplified to represent six generalized hydrogeologic units in the study area: (1) an uppermost alluvial and delta aquifer, (2) an upper confining unit, (3) an intermediate delta aquifer, (4) a lower confining unit, (5) an older buried delta aquifer, and (6) bedrock. The extent of model layers 1–5 was determined based on the mapped extent of young and old alluvium in Cambodia [5] or the extent of the delta as simulated by Minderhoud et al. (2017) in Vietnam. Model layer 6 (bedrock) was present throughout the entire model domain either beneath the alluvial and delta aquifer or at the land surface in the northern part of the study area. The top of the model was assigned based on the mean land-surface altitude in each model cell [6,7], and the bottom of each layer was calculated based on the assigned layer thickness, where active. All hydrogeologic units included in the proof-of-concept groundwater model were represented with generic and uniform thicknesses and hydraulic properties (Table S1).

Model stresses were applied for steady-state (pre-1990) and transient (water years 1991–2010) conditions. Stresses for the transient part of the model were applied such that four stress periods were used per year of the simulation. The stress periods were selected so that dry and wet seasons in the Mekong River Basin were each represented by two transient stress periods per year: (1) dry season *A* (Nov. 1–Jan. 31), (2) dry season *B* (Feb. 1–Apr. 30), (3) wet season *A* (May 1–Jul. 31), and (4) wet season *B* (Aug. 1–Oct. 31). Dry and wet seasons were determined based on Le (2020, Fig 2), and the three-month long stress periods were used to differentiate between periods of flood and drought that typically occur in a 12-month period [8]. Meters and days were used as the spatial and temporal units of discretization in the model, respectively.

Stresses simulated in the proof-of-concept groundwater model were recharge from precipitation, groundwater evapotranspiration, subsurface groundwater inflow and outflow, and streamflow, which allowed for the representation of the interaction between groundwater and surface water (Fig S1). Recharge from precipitation was simulated with the Recharge (RCH) Package; groundwater evapotranspiration was simulated using the Evapotranspiration (EVT) Package; subsurface groundwater inflow and outflow was simulated using the Specified Head (CHD) Package; and streamflow was simulated using the Streamflow Routing (SFR) Package.

Time-variable input for recharge and streamflow for the baseline and scenario simulations were applied at three-month intervals, and the temporal variations were consistent with the transient model stress periods. Steady-state stresses were applied such that they represented the mean rates of streamflow for October 1990 and mean annual water year recharge rate for the simulation period (water years 1991–2010). Recharge and evapotranspiration were applied in the model using spatially uniform values. For the baseline simulation, recharge was applied at an assumed rate of 16% of precipitation in Cambodia (Fig S2) [9]. Maximum potential evapotranspiration was 1.65 meters per year (m/yr) and the evapotranspiration extinction depth was 2.5 m [10]. Specified groundwater head applied to the CHD Package for cells representing groundwater inflow and outflow was assigned generically as 1 m below land surface altitude in areas where cross-boundary groundwater flow was most likely to occur. Groundwater head applied to the CHD Package for cells adjacent to the ocean was assigned as 0 m. Input streamflow for the SFR Package was applied at Phnom Penh, Cambodia (Fig S3) and was specified as the mean rate for each stress period based on streamflow at the Chroy Changvar streamgage that was calculated using a rating curve and daily stream stage observations [2]. The Bassac River bifurcates streamflow from the Mekong River in the model area downstream of Phnom Penh, Cambodia (Fig S1). Five percent of streamflow was assumed to bifurcate to the Bassac River from the Mekong River during each model stress period.

The proof-of-concept groundwater model was also used to simulate a 20-year hypothetical scenario, constructed to reflect the MRC climate scenario C3 [3]. The C3 climate scenario represents a 20-year period with 0.21 m of sea level rise in conjunction with drier conditions. Model inputs adjusted for the scenarios were the constant head at the ocean, streamflow, and recharge. Sea level rise was simulated by interpolating 0.21 m of rise throughout the 20-year period and applied to the constant head cells at the southwest model boundary [3]. Streamflow and recharge for the scenario simulation were based on estimates from the Mekong River Commission (2017, table 3-3) and Perera et al. (2017, Fig 5), respectively. Estimated streamflow and recharge at the end of the transient 20-year C3 dry scenario simulation period were linearly interpolated from the start of the simulation period and averaged to be representative of the three-month long stress periods used in the scenario simulation. The monthly percent change in precipitation and streamflow at the end of the 20-year C3 dry climate scenario were used to calculate inputs for the groundwater model scenario stress periods (Table S2). In the C3 dry climate scenario simulation, recharge in both the dry and wet seasons was lower compared to baseline conditions, but streamflow was typically higher in the dry season compared to baseline conditions and lower in wet season compared to baseline conditions (Table S2).

The outputs from the scenario simulation were used to calculate the change in groundwater levels relative to the baseline simulation. Transient groundwater level changes were calculated for the end of a transient stress period for the model layer from which the baseline water levels were obtained by subtracting the simulated groundwater levels in the uppermost non-dry model layer of the scenario simulation from the simulated groundwater levels in the same layer of the baseline simulation for equivalent model stress periods. The resulting groundwater level changes were then normalized by dividing the calculated groundwater level change (in m) by the saturated thickness of the layer (in m) in the baseline simulation and multiplied by a factor equivalent to the layer hydraulic conductivity (in meters per day [m/d]) divided by 50 m/d. The hydraulic conductivity factor adjusts for the largest hydraulic conductivity assigned to a layer in the model (50 m/d).

In general, normalized transient groundwater level changes were lower (groundwater level declines) in the C3 dry climate scenario compared to the baseline simulation. This result is indicated by the larger area of positive (+) numbers in Figs.S4*A*–*B* compared to negative (−) numbers. The greatest normalized groundwater level changes were simulated in the northwestern part of the model area in the delta and alluvial aquifers in Kampong Speu, Takev, and Kampot provinces in Cambodia where the variability in the land surface altitude is greatest among neighboring model cells. Normalized groundwater level declines for the C3 dry climate scenario were generally smaller in the dry season when compared to the wet season. While greater normalized groundwater level declines were simulated in the wet season for the C3 dry climate scenario, some increased groundwater levels were simulated beneath the Mekong and Bassac Rivers in the dry season because the streamflow was greater compared to the baseline simulation.

**Disclaimer:** Any use of trade, firm, or product names is for descriptive purposes only and does not imply endorsement by the U.S. Government.

**References**

1. Langevin CD, Hughes JD, Banta ER, Niswonger RG, Panday, Sorab, et al. Documentation for the MODFLOW 6 Groundwater Flow Model: U.S. Geological Survey Techniques and Methods. Book 6: Modeling Techniques. 2017. p. 197. Available: https://doi.org/10.3133/tm6A55.

2. Davis KW. MODFLOW 6 Proof-of-concept groundwater model for the Mekong River Basin between Phnom Penh, Cambodia and Cao Lanh, Vietnam. U.S. Geological Survey; 2023. Available: https://doi.org/10.5066/P9JGIWS4.

3. Mekong River Commission. Interactive report for the Council Study: Findings and Recommendations. Vientiane, Lao PDR: Mekong River Commission Secretariat; 2019 Sep. doi:10.52107/mrc.ajg58l

4. Minderhoud PSJ, Erkens G, Pham VH, Bui VT, Erban L, Kooi H, et al. Impacts of 25 years of groundwater extraction on subsidence in the Mekong delta, Vietnam. Environ Res Lett. 2017;12: 064006. doi:10.1088/1748-9326/aa7146

5. Open Development Cambodia. Geology of Cambodia. In: Open Development Mekong [Internet]. 2006. Available: https://data.opendevelopmentmekong.net/dataset/geology-of-cambodia-2006

6. Danielson JJ, Gesch DB. Global Multi-resolution Terrain Elevation Data 2010 (GMTED2010). Reston, Virginia: USGS; 2011 p. 26.

7. Minderhoud PSJ, Coumou L, Erkens G, Middelkoop H, Stouthamer E. Mekong delta much lower than previously assumed in sea-level rise impact assessments. Nat Commun. 2019;10: 3847. doi:10.1038/s41467-019-11602-1

8. Le AT. Water balance for agriculture production in the dry seasons of the Mekong River delta in Vietnam. Vietnam J Sci Technol Eng. 2020;62: 56–61. doi:10.31276/VJSTE.62(3).56-61

9. The World Bank. Download Data. In: Climate Change Knowledge Portal For Development Practioners and Policy Makers [Internet]. May 2021. Available: https://climateknowledgeportal.worldbank.org/download-data

10. MOWRAM. Study of Groundwater Resources in Kong Pisei & Basedth District of Kampong Speu Province, Cambodia. WAPCOS Limited; 2018 p. 763.

11. Perera EDP, Sayama T, Magome J, Hasegawa A, Iwami Y. RCP8.5-Based Future Flood Hazard Analysis for the Lower Mekong River Basin. Hydrology. 2017;4: 55. doi:10.3390/hydrology4040055
