# Supplementary material for: An integrated framework for examining groundwater vulnerability in the Mekong River Delta region
Source: PLoS One. 2023 Oct 20;18(10):e0292991. doi: 10.1371/journal.pone.0292991 (PMC10588840; doi:10.1371/journal.pone.0292991)
Supplement: S1 Fig — (DOCX) [file pone.0292991.s002.docx]

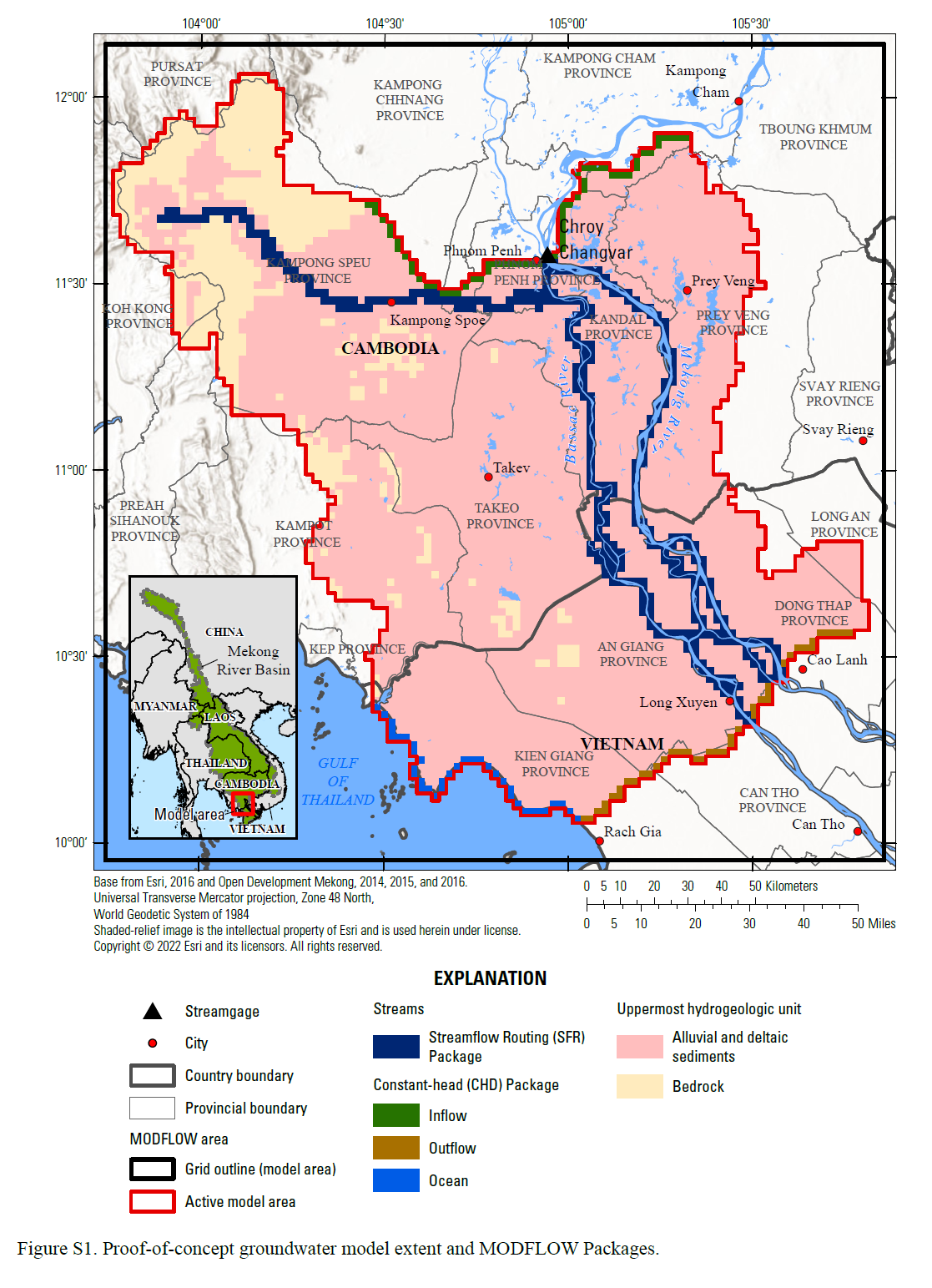


S1 Fig. Proof-of-concept groundwater model extent and grid cells linked to specific MODFLOW Packages.
