# Supplementary material for: An integrated framework for examining groundwater vulnerability in the Mekong River Delta region
Source: PLoS One. 2023 Oct 20;18(10):e0292991. doi: 10.1371/journal.pone.0292991 (PMC10588840; doi:10.1371/journal.pone.0292991)
Supplement: S3 Fig — (DOCX) [file pone.0292991.s004.docx]

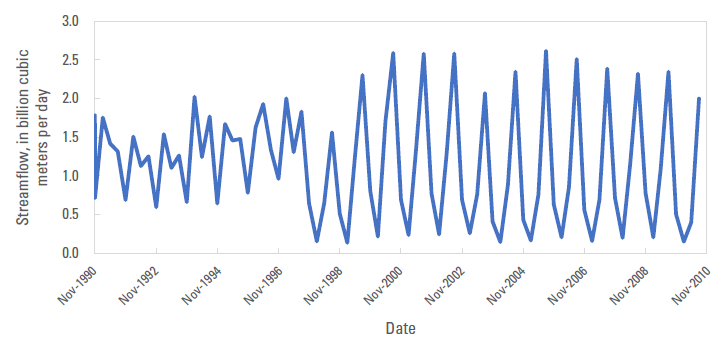


S3 Fig. Input streamflow at Phnom Penh, Cambodia for the baseline proof-of-concept groundwater model.
