# Supplementary material for: An integrated framework for examining groundwater vulnerability in the Mekong River Delta region
Source: PLoS One. 2023 Oct 20;18(10):e0292991. doi: 10.1371/journal.pone.0292991 (PMC10588840; doi:10.1371/journal.pone.0292991)
Supplement: S4 Fig — Relative groundwater level changes after 20 years of drier climate (C3) with sea-level rise for the A, wet season and B, dry season. Positive values indicate areas of groundwater level declines and negative values indicate increased groundwater levels. (DOCX) [file pone.0292991.s005.docx]

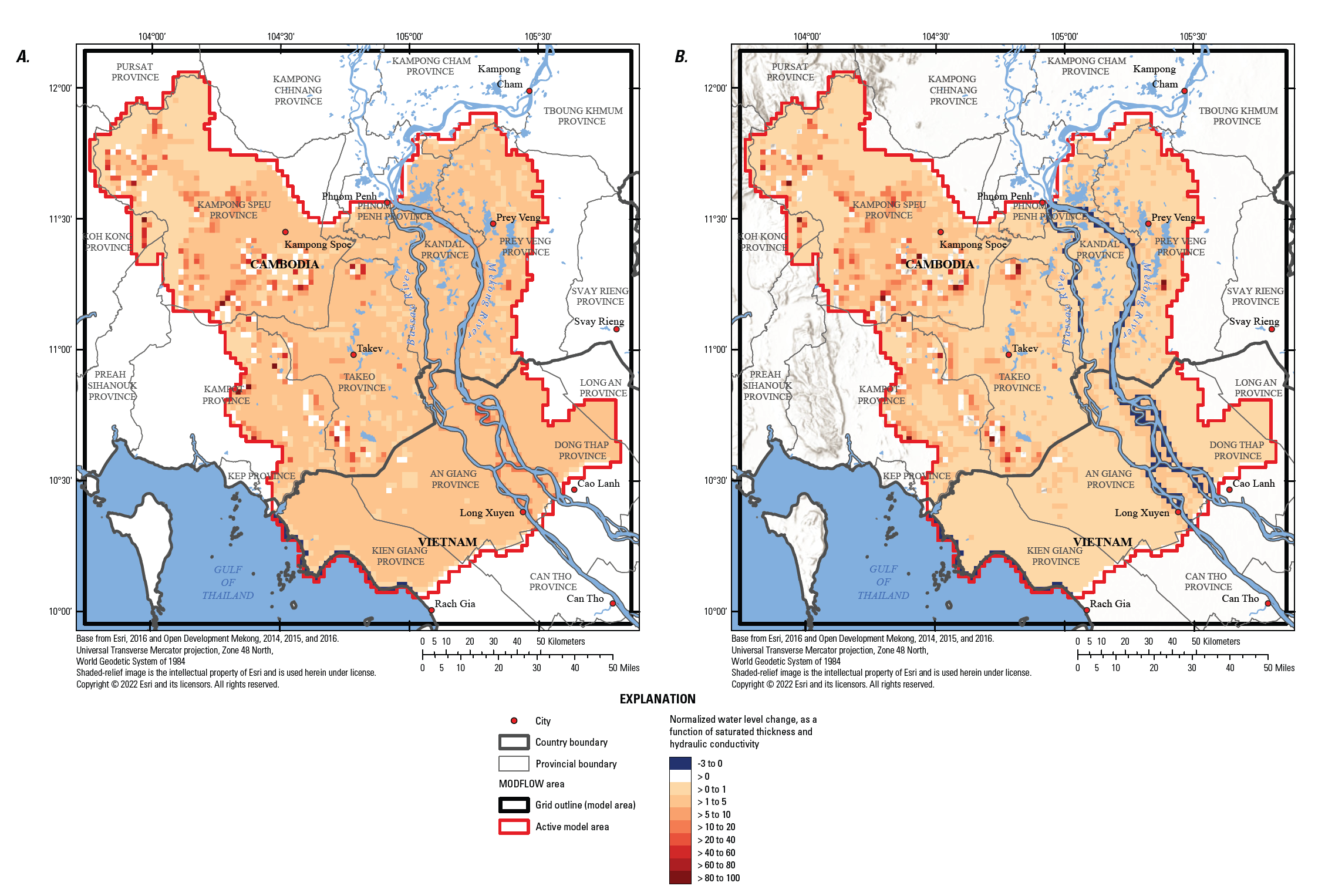


*S4 Fig. Relative groundwater level changes after 20 years of drier climate (C3) with sea-level rise for the* ***A****, wet season and* ***B****, dry season. Positive values indicate areas of groundwater level declines and negative values indicate increased groundwater levels.*
