# Supplementary material for: An integrated framework for examining groundwater vulnerability in the Mekong River Delta region
Source: PLoS One. 2023 Oct 20;18(10):e0292991. doi: 10.1371/journal.pone.0292991 (PMC10588840; doi:10.1371/journal.pone.0292991)
Supplement: S1 Table — (DOCX) [file pone.0292991.s006.docx]

S1 Table. Summary of hydrogeologic units in the groundwater model.

| **Layer** | **Description** | **Thickness (m)** | **Hydraulic conductivity (m/d)** | | **Specific storage (m^-1^)** | **Specific yield (%)** |
| --- | --- | --- | --- | --- | --- | --- |
|  |  |  | **Horizontal** | **Vertical** |  |  |
| 1 | Uppermost alluvial and delta aquifer | 12 | 50 | 15 | 1.10x10^-4^ | 15 |
| 2 | Upper confining unit | 2 | 0.1 | 0.005 | 5.50x10^-4^ | 1 |
| 3 | Intermediate delta aquifer | 4 | 15 | 3 | 8.25x10^-5^ | 10 |
| 4 | Lower confining unit | 2 | 0.1 | 0.005 | 5.50x10^-4^ | 1 |
| 5 | Older buried delta aquifer | 10 | 5 | 0.5 | 5.50x10^-5^ | 5 |
| 6 | Bedrock | 70 | 2.5 | 0.25 | 5.50x10^-6^ | 3 |
