# Supplementary material for: An integrated framework for examining groundwater vulnerability in the Mekong River Delta region
Source: PLoS One. 2023 Oct 20;18(10):e0292991. doi: 10.1371/journal.pone.0292991 (PMC10588840; doi:10.1371/journal.pone.0292991)
Supplement: S2 Table — Positive values indicate an increase from baseline. (DOCX) [file pone.0292991.s007.docx]

S2 Table. Monthly percent change in recharge and streamflow applied to the end of the 20-year C3 climate scenario (Mekong River Commission, 2019), drier climate with sea level rise. Positive values indicate an increase from baseline.

| **Month** | **Model season** | **Change, after 20 years of drier climate (percent)** | |
| --- | --- | --- | --- |
|  |  | **Precipitation^1,2^** | **Streamflow^3^** |
| November | Dry A | −22.9 | −4.9 |
| December |  | −77.8 | 15 |
| January |  | −100.0 | 33.1 |
| February | Dry B | −58.3 | 37.3 |
| March |  | −25.0 | 24.6 |
| April |  | −14.5 | 10.2 |
| May | Wet A | −8.9 | −24.8 |
| June |  | −9.1 | −28.3 |
| July |  | −8.5 | −28.3 |
| August | Wet B | −11.0 | −18.4 |
| September |  | −6.8 | −9.4 |
| October |  | −5.6 | −10.5 |
| ^1^ Recharge was calculated as 16% of precipitation in the study area. | | | |
| ^2^ Based on Perera et al. (2017, Fig 5). | | | |
| ^3^ Based on Mekong River Commission (2017, table 3-3). | | | |
